# Molecular Epidemiology of Omicron CH.1.1 Lineage: Genomic and Phenotypic Data Perspective

**DOI:** 10.7759/cureus.53496

**Published:** 2024-02-03

**Authors:** Yasir Mohammed A Al Qurashi, Jawaher A Abdulhakim, Samia S Alkhalil, Maymuna Alansari, Renad Almutiri, Rageed Alabbasi, Manal S. Fawzy

**Affiliations:** 1 Department of Medical Laboratory, College of Applied Medical Sciences, Taibah University, Yanbu, SAU; 2 Department of Clinical Laboratory Sciences, College of Applied Medical Sciences, Shaqra University, Alquwayiyah, SAU; 3 Department of Biochemistry, Unit of Medical Research and Postgraduate Studies, Faculty of Medicine, Northern Border University, Arar, SAU

**Keywords:** s: k444t, s: f486s, s: r346t, s: l452r, ba.2.75, ch.1.1 lineage

## Abstract

Background: The Omicron variant (B.1.1.529 lineage) of SARS-CoV-2 represents a substantial global health challenge due to its high transmissibility and potential resistance to immunity from vaccines or previous infections. Among the rapidly evolving Omicron lineages, the BA.2.75 and the emerging CH.1.1 have garnered attention. While BA.2.75 is marked by mutations that may enhance immune evasion, CH.1.1 is distinguished by the S: L452R mutation, linked to increased pathogenicity and transmission. Initially identified in India by the end of 2021, these variants have exhibited global dissemination, signaling an urgent need to track and analyze their progression.

Methods: In this study, the genomic and geographical distribution data of CH.1.1 were collected from the Global Initiative on Sharing Avian Influenza Data (GISAID), PANGOLIN, CoV-Spectrum, and NextStrain databases. Due to the unavailability of epidemiological and genomic data of the CH.1.1 lineage, PubMed and ScienceDirect were used as sources of the phenotypic data of the lineage variations. Amino acid variations utilized in the data mining included S: R346T, S: K444T, S: L452R, and S: F486S.

Results: The current epidemiological data indicate that CH.1.1 is more likely to become one of the dominant spreading lineages in the United Kingdom, New Zealand, Australia, and the United States based on a 32% growth advantage, present CH.1.1 lineage cases number, and the amino acid variation's impact.

Conclusion: A significant increase in the newly detected lineage CH.1.1 is highly anticipated. The rise in the detected sequences number from 13,231 on January 21, 2023, to 23,181 on February 6, 2023, supports the prediction and growth advantage of the lineage detected cases. Increases in viral transmissibility caused by higher affinity to ACE2 receptors and immune evasion are deduced from amino acid variations analyzed in the study.

## Introduction

The recent emergence of the Omicron variant, or Pango lineage B.1.1.529, of the severe acute respiratory syndrome coronavirus-2 (SARS-CoV-2) has raised significant global public health concerns [1]. The variant declared a variant of concern (VOC) by the World Health Organization on November 25, 2021, is characterized by its enhanced transmissibility and lower susceptibility to neutralizing antibodies from vaccination or past infection [2]. In a short period, the Omicron variant has undergone rapid genomic evolution, yielding five initial lineages (BA.1.1, BA.2, BA.3, BA.4, and BA.5), including the BA.2.75 lineage, bearer of multiple amino acid variations in the spike protein potentially enhancing immune evasion and ACE2 binding affinity [3].

First detected in India at the end of 2021, this lineage has since spread globally to nations such as Australia, Canada, the United Kingdom, the USA, and Japan, amassing upwards of 90,778 sequenced samples by early January 2023, with prevalence rising particularly sharply during the middle months of 2022 [4]. In addition to BA.2.75, another sub-lineage, CH.1.1, was first reported from sequences submitted from Australia, Austria, and the United States between August and September 2022. CH.1.1 cases were soon detected in several countries, with a prevalence thought to be increasing [5]. Significant by its delta variant-like S: L452R variation, it is considered a highly pathogenic and transmissible lineage [6]. This study aims to analyze the genomic and phenotypic data of the Omicron CH.1.1 lineage; investigate the geographical distribution and growth advantage of CH.1.1 in multiple countries; assess the impact of amino acid variations (such as S: R346T, S: K444T, S: L452R, and S: F486S) on transmissibility and immunity; compare the characteristics of the CH.1.1 lineage with other Omicron lineages, particularly BA.2.75; and provide insights into the potential rise of CH.1.1 as a dominant spreading lineage.

## Materials and methods

**Figure 1:**
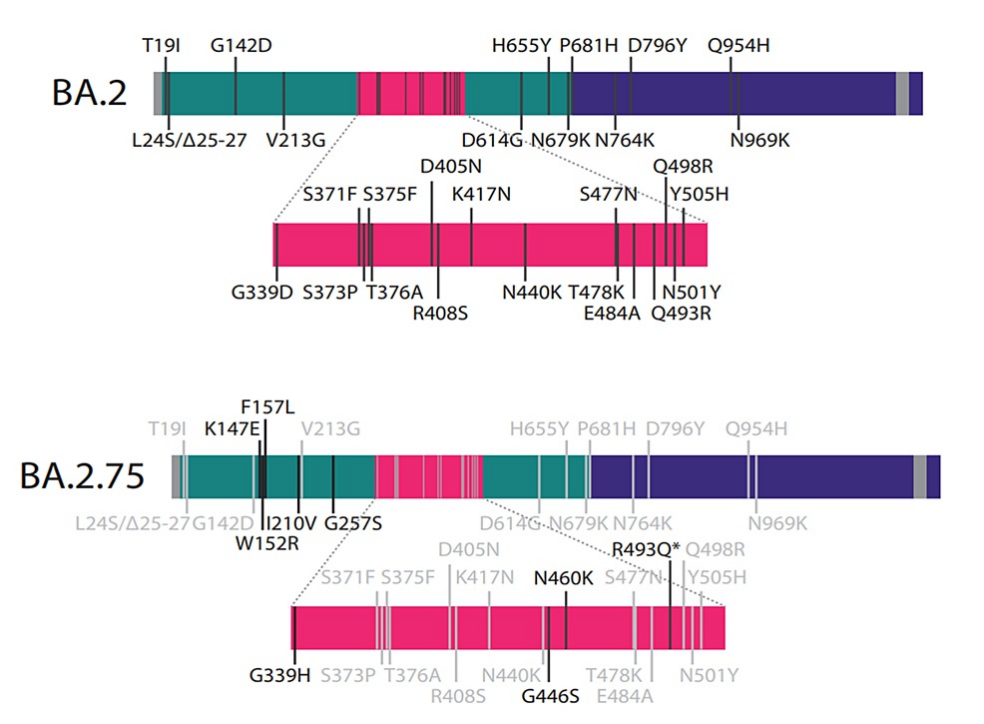
Amino acid variations on the S protein-encoding region of Omicron lineages BA.2 and BA.2.75. * indicates reversion variation (image credits: [13]).

The SARS-CoV-2 Omicron variant is also known as Pango lineage B.1.1.529 by Phylogenetic Assignment of Named Global Outbreak Lineages (PANGOLIN) [7]. It was classified by the WHO as a VOC on November 25, 2021 [8]. This designation was due to its high transmissibility and low susceptibility to neutralizing antibodies (nAbs) produced by the viral infection or vaccination [9]. Omicron has shown a high capability of continuous genomic evolution in a short period of time. This resulted in the emergence of Omicron's first five lineages: BA.1.1, BA.2, BA.3, BA.4, and BA.5 [10,11]. BA.2.75 is a lineage of BA.2, showing an increasing prevalence in the second half of 2022. It raised concerns due to its acquisition of multiple amino acid variations on the spike (S) protein, rapid growth, and extensive geographical distribution [12]. Compared to BA.2, BA.2.75 carries nine additional variations in the S protein: K147E, W152R, F157L, I210V, G257S, G339H, G446S, N460K, and R493Q (Figure 1) [13].

**Figure 2:**
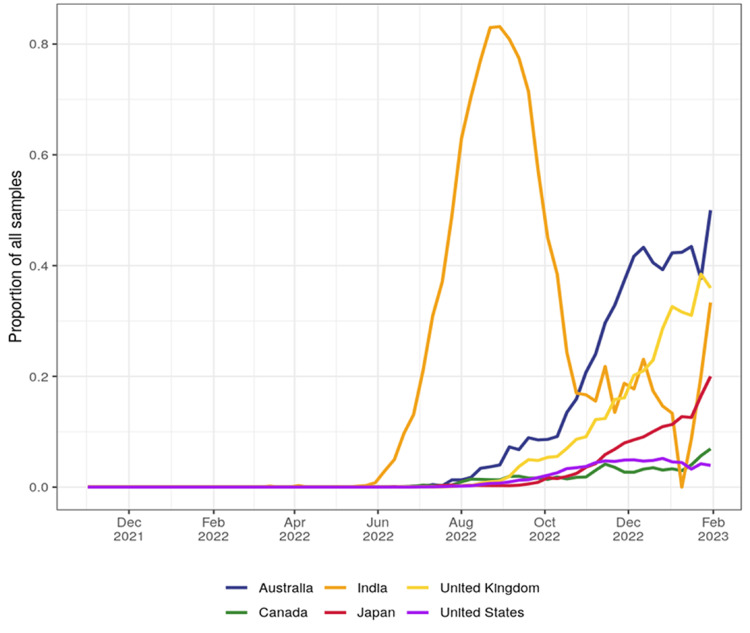
The growth rate of BA.2.75 in India, Australia, the United Kingdom, Japan, the USA, and Canada. BA.2.75 rapidly grew in India, with a significant increase in the curve observed at 83.89% by the end of August 2022 (image credits: [16]).

The last four variations (state them) are likely to result in immune evasion and increased binding affinity to angiotensin-converting enzyme 2 (ACE2) [14]. BA.2.75 lineage was first detected in India on December 31, 2021 [15], and spread rapidly to other countries, including Australia, Canada, the United Kingdom, the USA, and Japan [12]. The total samples sequenced of BA.2.75 are 90,778 up to January 9, 2023. Based on the CoV-Spectrum data, a significant rise of BA.2.75 spread was noticed in July and August 2022 (Figure 2) [16].

**Figure 3:**
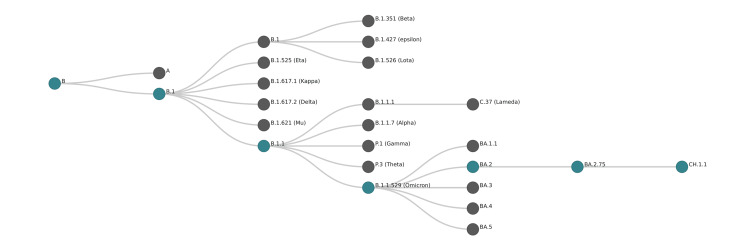
Lineage CH.1.1 ancestral illustrative map, showing the gradual arrangement of the lineages, starting from the ancestor Omicron to the CH.1.1 descendant. The map was constructed using the NextStrain clades schema template in the CodeSandbox platform.

Similar concerns are also rising with the emergence of the CH.1.1 lineage (an alias of BA.1.1.529.2.75.3.4.1.1.1.1) [7]. The lineage is a descendant of CH.1 (an alias of B.1.1.529.2.75.3.4.1.1.1) [7] and is a descendant of BA.2.75 (Figure 3). The first report of the CH.1 lineage sequence included three sequences from Australia, Austria, and the USA in August and September of 2022 (the software package for assigning SARS-CoV-2 genome sequences to global lineages; GitHub 2022). According to the Global Initiative on Sharing All Influenza Data (GISAID), the earliest date of CH.1.1 lineage detection was on July 27, 2022 (EPI_ISL_16557370) [15]. By the middle of September 2022, variable positive CH.1.1 cases were detected in multiple countries [16]. The emerging CH.1.1 lineage is defined only by the variation S: L452R [7], also found in the Delta variant, the most pathogenic and highly transmissible lineage worldwide [17].

The current statistics of the CH.1.1 rate indicate an increase in lineage prevalence, and it is estimated to advance to the fifth highest proportion among the variants spreading in the USA (Table 1) [18]. This study was conducted as a molecular epidemiological analysis to investigate the current CH.1.1 geographical lineage distribution, genomic and phenotypic data, and amino acid variations.

**Table 1:** The statistics of the CH.1.1 rate in the USA during the study period. WHO: World Health Organization, Lineage: A lineage is a group of closely related viruses with a common ancestor, VOC: Variant of concern, VBM: Variants being monitored, 95%PI: 95% Prediction intervals (data sources: [18]).

WHO label	Lineage #	US Class	% Total	95%PI
Omicron	XBB.1.5	VOC	66.4%	59.8-72.5%
	BQ.1.1	VOC	19.9%	16.2-24.1%
	BQ. 1	VOC	7.3%	5.8-9.0%
	XBB	VOC	2.3%	1.8-2.8%
	CH.1.1	VOC	1.6%	1.2-2.0%
	BN.1	VOC	1.1%	0.9-1.4%
	BA.5	VOC	0.5%	0.4-0.7%
	BF.7	VOC	0.5%	0.4-0.6%
	BA.5.2.6	VOC	0.2%	0.1-0.2%
	BA.2	VOC	0.1%	0.1-0.2%
	BF.11	VOC	0.1%	0.1-0.1%
	BA.2.75	VOC	0.0%	0.1-0.1%
	BA.4.6	VOC	0.0%	0.0-0.0%
	BA.2.75.2	VOC	0.0%	0.0-0.0%
	B.1.1.529	VOC	0.0%	0.0-0.0%
	BA.4	VOC	0.0%	0.0-0.0%
	BA.1.1	VOC	0.0%	0.0-0.0%
	BA.2.12.1	VOC	0.0%	0.0-0.0%
Delta	B.1.617.2	VBM	0.0%	0.0-0.0%
Other	Other		0.1%	00-0.1%

**Figure 4:**
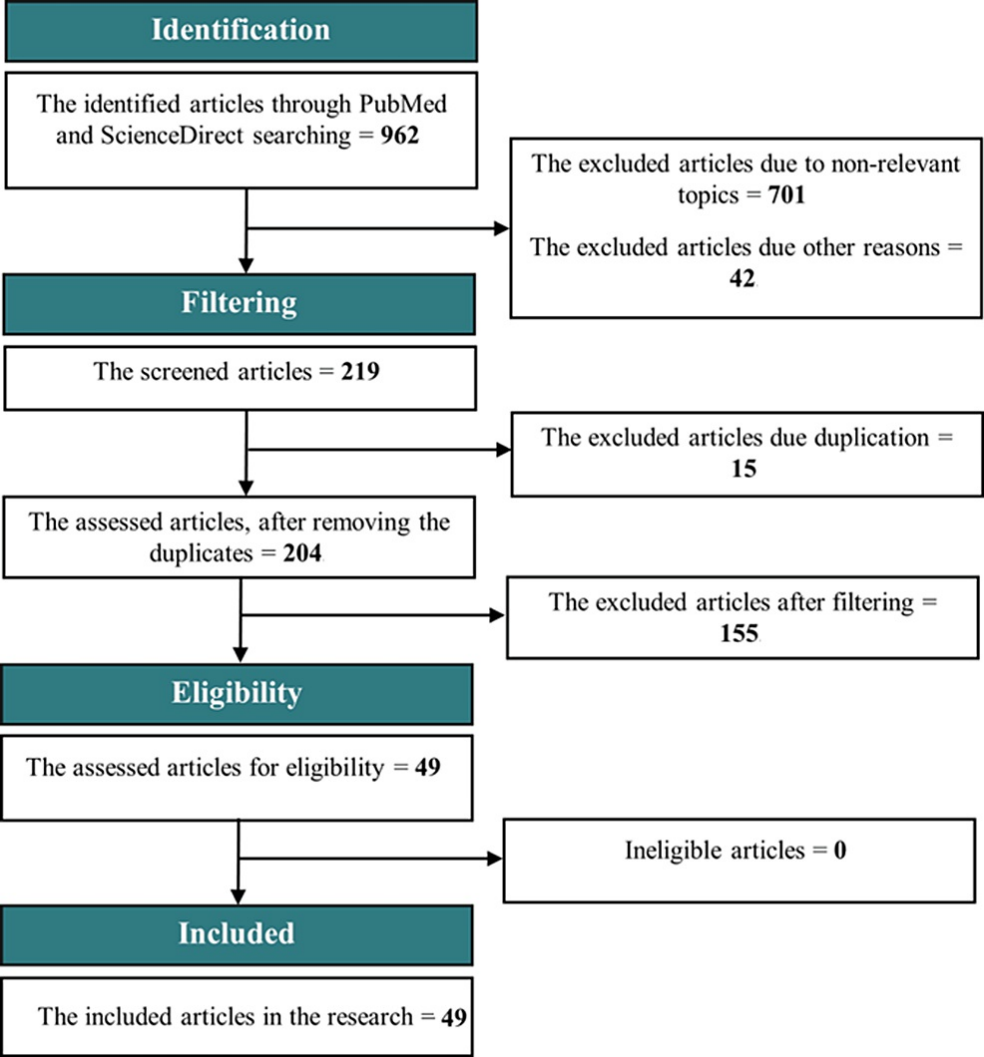
Genomic and phenotypic data mining flow chart.

The genomic and geographical distribution data of CH.1.1 in this study was mined from GISAID, PANGOLIN, CoV-Spectrum, and NextStrain databases. Due to the unavailability of published studies of lineage CH.1.1 in PubMed and ScienceDirect databases, amino acid variations (R346T, K444T, L452R, and F486S) were searched for related articles published from 2020 to 2023 according to the data mining flow chart (Figure 4). Duplicate articles, non-relevant topics, or articles not meeting the date range were excluded. Filters used in the search included 'free full text,’ 'English language,' and 'associated data.' This study has been in progress from January 7 to February 14, 2023.

The researchers used a combination of specific and broad terms, often with Boolean operators (AND, OR, NOT), to refine the search while retrieving the epidemiological and genomic data (Table 2). Adapting search terms according to the specificity required by the database or the information sought was essential to obtaining relevant and comprehensive data.

**Table 2:** Search terms and strategies utilized in genomic databases and scientific literature searches.

No.		Search terms
1.	Variant and Lineage-Specific Terms:	"Omicron variant" "CH.1.1 lineage" "SARS-CoV-2 variant CH.1.1" "B.1.1.529 sublineage" "Omicron subvariant"
2.	Genomic and Mutation-Specific Terms:	"S gene mutations" "spike protein mutations" "R346T" "K444T" "L452R" "F486S" "Nucleotide changes" "Amino acid substitutions"
3.	Geographical Distribution and Prevalence:	"Global distribution of Omicron CH.1.1" "Prevalence of CH.1.1" "Omicron CH.1.1 cases" "Geographic spread of SARS-CoV-2 variants"
4.	Temporal Data Retrieval:	"2023 Omicron epidemiology" "SARS-CoV-2 sequence data [specific months/years]" "COVID-19 variant surveillance"
5.	Phenotypic and Clinical Outcomes:	"Omicron clinical presentation" "COVID-19 Omicron symptoms" "CH.1.1 phenotype characterization"
6.	Database and Science Repositories:	Site-specific commands, i.e., for GISAID: "hCoV-19 / Omicron / Lineage / CH.1.1" "Pangolin COVID-19 Lineage Assigner" "CoV-Spectrum variant analysis" "NextStrain genomic epidemiology of SARS-CoV-2"
7.	Combinations Using Boolean Operators:	"(Omicron OR B.1.1.529) AND (CH.1.1 OR 'sublineage') AND mutations" "(Omicron CH.1.1) NOT (Delta OR Alpha) to exclude other variants "COVID-19 AND (variant OR mutation) AND 'clinical outcomes'"
8.	Review and Study Type Filters:	"Review articles" "Epidemiological studies" "Genomic sequencing studies"

## Results

**Figure 5:**
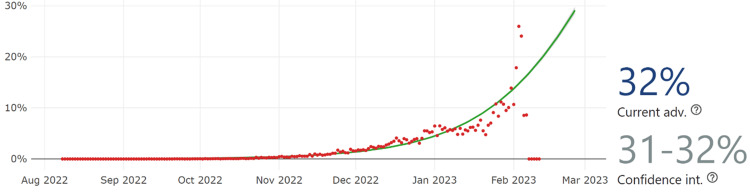
The current CH.1.1 growth advantage is 32% with a confidence interval of 32-33% worldwide. Note: The green line represents the logistic fit of the curve, while the red dots are the estimated daily proportion (image credits: [16]).

Considering the CoV-Spectrum data, the total number of sequences is 23,181 cases. With the current estimation of CH.1.1, the growth advantage is 32%, indicating an increase in the detection percentage of the variant reaching 32% among all variants detected in COVID-19 patients,” as shown in Figure 5.

**Figure 6:**
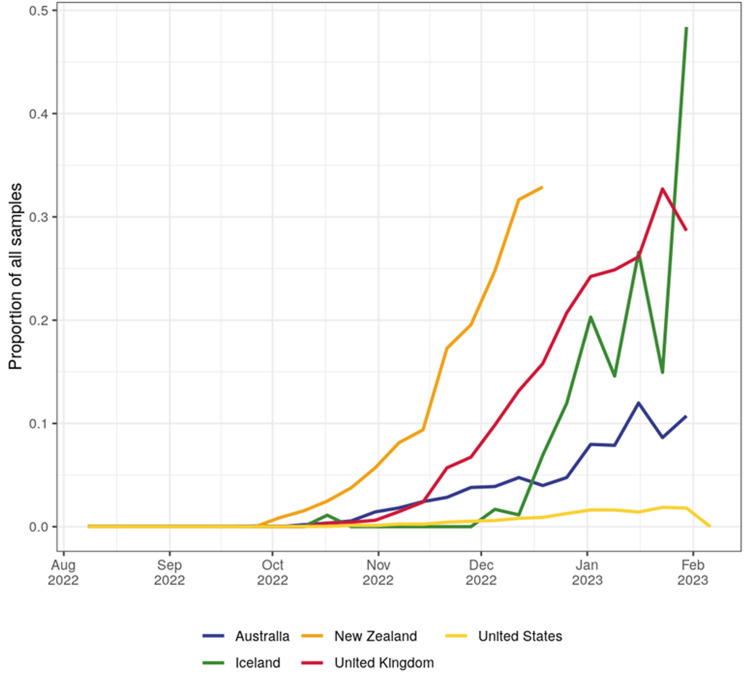
The graph illustrates CH.1.1 growth in the United Kingdom, New Zealand, Iceland, Australia, and the USA until February 2023. Image credits: [16]

It has grown enormously in Europe, with a high number of submitted sequences of 16,465. The countries where this lineage grows are the United Kingdom, New Zealand, Iceland, Australia, and the USA until February 2023 (Figure 6).

**Figure 7:**
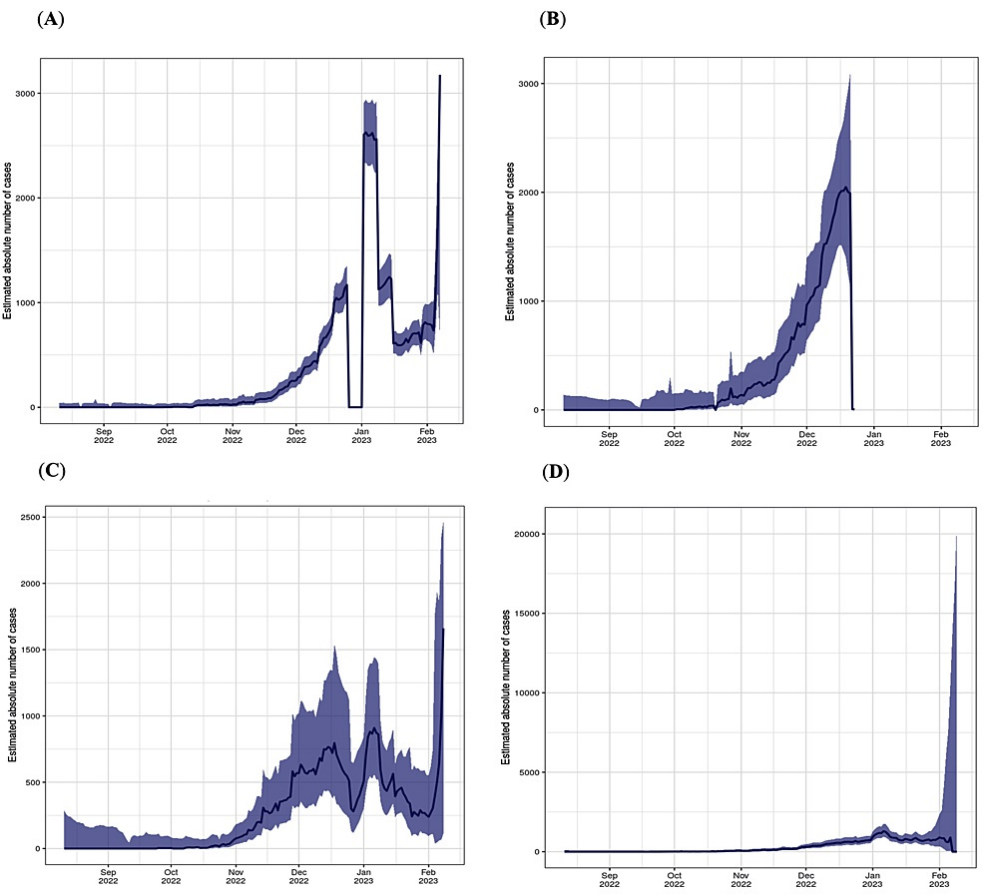
The case number of CH.1.1 infections in the United Kingdom, New Zealand, Australia, and the USA. (A) In the United Kingdom, where there are 3,176 cases as of February 2, 2023. (B) The highest number of submitted cases in New Zealand was 2,049 on December 23, 2022. (C) The curve of Australia shows a fluctuation in the number of cases. On February 7, 2023, the most recent update was 996 cases. (D) The highest number of estimated cases in the USA was on January 6, 2023 (image credits: [16]).

CH.1.1 is predicted to become one of the dominant lineages in these countries according to the following data. On February 7, 2023, the number of cases in the United Kingdom appeared to be 2,176, as the curve in Figure 7a shows, with an estimated growth advantage of 35% in the middle of February 2023. The growth of the lineage in New Zealand has dramatically increased since the beginning of November 2022. According to CoV-Spectrum, CH.1.1 in New Zealand is estimated to have a growth advantage at 45% in early January of 2023. Figure 7b illustrates the increasing number of cases in New Zealand from December 2022 up to 2049 case. On the other hand, the growth advantage of CH.1.1 in the USA is predicted to be 28% by February 21, 2023. Other countries are predicted to have a high growth advantage by the beginning of 2023, such as Iceland, with 53% (confidence interval 41-65%) by the middle of February 2023, the highest estimated growth of all the reported countries [16].

## Discussion

CH.1.1 is classified as a VOC by the Centre for Disease Prevention and Control [18] due to its rapid spread and the significant increase in growth rate every one to two weeks [19]. The rapid transmissibility of CH.1.1 may result from the defining amino acid variation S: L452R and other variations, including S: R346T, S: K444T, and S: F486S, located on the receptor-binding domain (RBD). The variations on these residues are commonly capable of evading antibodies and have a high binding affinity to ACE2 [20]. In addition to BA.2.75 spike protein variations, CH.1.1 is highly suspected of evading nAbs generated by a vaccine or infection [19]. Furthermore, the four variations were detected in BQ.1.1 lineage, except for S: F486S, which has a substitution at the same position but from phenylalanine to valine (F486V) [20]. It demonstrates high therapeutic antibody evasion and a sufficient binding affinity to ACE2. However, the impact of these variations was detected earlier in other lineages, for which it is expected to have similar characteristics [18].

The S protein must sufficiently bind to the ACE2 receptor to gain an advantage in the transmission. The amino acid-defining variation S: L452R has a high affinity to the ACE2 receptor, while the other variations have a sufficient affinity to the ACE2 receptor. In contrast, S: F486S has reduced the binding affinity of the ACE2 receptor, while K444T has no significant impact. The significant immune evasion effect of S: R346T and S: L452R has been detected in BA.2.75, in addition to S: K444T in BQ.1, BQ.1.1, BA.5.6.2, and BA.4.6.1. Additionally, S: F486S was found in XBB and BA.2.75. XBB is one of the dominant lineages nowadays; the presence of similar variations may indicate a similar impact [17, 21]. CH.1.1 is defined by S: L452R, while the other defining variations are for its parental strains. For example, BM.4.1 is defined by S: F486S, BM.4.1.1 by S: R346T, and S: K444T substitution for CH.1 [1].

According to Outbreak.info, the noticeable early spread of the CH.1.1 infection occurred on September 19, 2022 [18], and on September 16, 2022, according to PANGOLIN [7]. The present analysis has revealed key patterns in the prevalence and growth advantage of the CH.1.1 lineage of SARS-CoV-2. Based on the CoV-Spectrum data, a total number of 23,181 cases were reported on February 7, 2023. Notably, the growth advantage of CH.1.1 is estimated to be 32%, suggesting rapid lineage propagation. European countries were significantly affected, contributing to 16,465 of the submitted sequences thus far. Furthermore, the United Kingdom, New Zealand, the USA, and Australia were contending with a dramatic rise in the prevalence of this particular lineage [18].

In the United Kingdom, the rate of growth was found to be steep, with the number of cases surging to 2,176 by the middle of February 2023, representing a 35% growth advantage. In New Zealand, reports indicate an efficient foothold of this lineage since November 2022, culminating in a high growth advantage of 45% in early January 2023 and leading to 2,049 reported cases by February 2023, as illustrated in Figure 7. The United States is also witnessing the steady rise of the CH.1.1 lineage, although at a slightly slower pace than the aforementioned countries. The growth advantage in this region is predicted to be 28% by February 21, 2023. Intriguingly, Iceland has emerged as a fast-growing hotspot of the CH.1.1 variant. With a predicted growth advantage of 53% by mid-February 2023, this country exhibits the highest estimated growth among all reported countries. This evidence underscores the formidable transmissibility of this lineage and portends potential challenges in global COVID-19 containment efforts [22].

It is pivotal to closely monitor the spread of the CH.1.1 variant to inform public health strategies that can mitigate its impact. Future studies should focus on the lineage's effect on vaccine efficacy, potential reinfection risks, and disease severity to facilitate comprehensive responses to this evolving pandemic [23].

The present work has several strengths, including the rigor of the methodologies employed, such as the use of multiple databases (GISAID, PANGOLIN, CoV-Spectrum, NextStrain) and a comprehensive literature review; discussion of the relevance and importance of the chosen amino acid variations (S: R346T, S: K444T, S: L452R, and S: F486S) in understanding the characteristics of the Omicron CH.1.1 lineage; explanation of why these variations were selected and how they contribute to the study's objectives; and comparison of the CH.1.1 lineage with other Omicron lineages, particularly BA.2.75, which would strengthen the study's contribution to the understanding of different variants. However, it is essential to acknowledge the potential limitations of our study as (a) data availability and completeness: the reliance on available genomic/epidemiological data, which may not capture all cases of the CH.1.1 lineage due to differences in national sequencing efforts, reporting lags, and under-reporting; (b) the rapid evolution of SARS-CoV-2: the data might quickly become outdated, and newer lineages or variants may emerge that could alter the prevalence and significance of CH.1.1; (c) sampling bias: the genomic sequences available in databases such as GISAID are not representative of the global population due to higher sequencing rates in certain regions; (d) the geographic concentration of data that might limit the generalizability of the findings to the global population; (e) phenotypic analysis constraints: the actual phenotypic data, such as clinical outcomes or in vitro studies, might be sparse or unavailable for new lineages such as CH.1.1 (this limits the ability to correlate genomic data with real-world virulence or clinical severity); (f) predictive modeling challenges: predictions based on the current spread and growth rate of CH.1.1 could be constrained by unforeseen factors such as public health interventions, changes in population behavior, or the emergence of immune escape variants; (g) lab validation: the impact of specific amino acid changes on viral function is anticipated based on previous knowledge but might require further laboratory confirmation to understand these effects on transmissibility and immune evasion; and (h) time-limited snapshot of the genomic and phenotypic landscape, which may not account for subsequent developments in the pandemic response or virus evolution.

## Conclusions

This molecular epidemiological study reports the current data of CH.1.1 lineage geographical distribution and the possible impact of its defining variation and other amino acid variations. The current study predicts a significant increase in the CH.1.1 lineage detection and suggests that it is more likely to rise among the dominant spreading lineages of COVID-19 cases in the United Kingdom, Australia, and New Zealand. This estimation is based on GISAID records of submitted sequences of the lineage from infected cases. The rise in the detected sequence number from 13,231 on 21 January 2023 to 23,181 on 6 February 2023 supports the prediction and growth advantage of the lineage detected cases. Geographical distribution of the viral lineage sequences suggests that CH.1.1 is more likely to become one of the dominant spreading lineages in the United Kingdom, New Zealand, Australia, and the USA based on 32% growth advantage and present CH.1.1 lineage cases number. Deduced phenotypic characteristics of CH.1.1, including the affinity of the viral lineage to neutralizing antibodies, support the estimated growth value of the lineage. It is worth noting that the current study prediction might be influenced by public health measures changes in these countries. Additionally, this prediction included developed countries with high rates of COVID-19 samples sequencing. In this sense, the study could not predict the current trend of the lineage in countries with low sequencing capacity or low-quality sequencing capacity.
